# ANNOUNCEMENTS & RESOURCES

**Published:** 2017-05-12

## World Sight Day

This year, the call to action we encourage you to use is “Make Vision Count”. In 2010, just over 28% of the world's population were affected by Myopia (shortsightedness). This is predicted to rise to 34% by 2020 and nearly 50% by 2050. In 2014, approximately 422 million people – or 8.5% of adults worldwide – were living with diabetes, compared to 108 million in 1980. Low- and middle-income countries account for approximately 75% of the global diabetes burden. Approximately one in three people living with diabetes have some degree of Diabetic Retinopathy (DR) and one in 10 will develop a vision-threatening form of the disease.

This World Sight Day, let's get the numbers out, so we know where we stand. Do write to **communications@iapb.org** if you want to receive World Sight Day promotional material like posters, balloons and ribbons.

You can download print quality logos for World Sight Day, here: **www.iapb.org/wsd17/promotional-material**

## Courses

**MSc Public Health for Eye Care, London School of Hygiene & Tropical Medicine** 10 fully funded scholarships available for Commonwealth Country Nationals. Course aims to provide eye health professionals with the public health knowledge and skills required to reduce blindness and visual disability in their setting. For more information visit: **www.lshtm.ac.uk/study/masters/mscphec.html** or email **romulo.fabunan@lshtm.ac.uk**

**Online International Eye Bank Qualification course** – Now accepting applications for the September intake of the Graduate Certificate and shorter Specialist Certificate. Apply and enquire via: **http://commercial.unimelb.edu.au/custom-education/courses/eyebankinggc**

## Subscriptions

Contact Anita Shah **admin@cehjournal.org**

### Subscribe to our mailing list

**web@cehjournal.org** or visit **www.cehjournal.org/subscribe**

### Visit us online

**www.cehjournal.org www.facebook.com/CEHJournal https://twitter.com/CEHJournal**

